# Association between abdominal obesity and depressive symptoms in Peruvian women aged 18–49 years: a sub-analysis of the Demographic and Family Health Survey 2018–2019

**DOI:** 10.1017/S1368980024000867

**Published:** 2024-04-12

**Authors:** Sharon Leon-Zamora, David Villarreal-Zegarra, Luciana Bellido-Boza

**Affiliations:** 1 Facultad de Ciencias de la Salud, Universidad Peruana de Ciencias Aplicadas, Lima, Peru; 2 Instituto Peruano de Orientación Psicológica, Lima, Peru; 3 Escuela de Psicología, Universidad Continental, Lima, Peru

**Keywords:** Obesity, Abdominal obesity, Depression, Patient Health Questionnaire, Women, Peru

## Abstract

**Objective:**

Abdominal obesity (AO) is characterised by excess adipose tissue. It is a metabolic risk that affects the physical and mental health, particularly in women since they are more prone to mental health problems like depression. This study investigated the association between AO and depressive symptoms in Peruvian women of reproductive age (18–49 years).

**Design:**

This is a cross-sectional observational study.

**Setting:**

Peruvian women population of reproductive age.

**Participants:**

We used data from the Peruvian Demographic and Family Health Survey (DHS) for 2018 and 2019 to assess 17 067 women for the presence of depressive symptoms (using the Patient Health Questionnaire (PHQ-9): cut-off score ≥ 10) and AO (measured by abdominal circumference; cut-off score ≥88 cm).

**Results:**

We observed a 64·55 % prevalence of AO and 7·61 % of depressive symptoms in the study sample. Furthermore, 8·23 % of women with AO had depressive symptoms (*P* < 0·05). Initially, women with AO appeared to have a 26 % higher risk of depressive symptoms compared with women without AO (*P* = 0·028); however, after adjustment for covariates, no statistically significant association was observed.

**Conclusions:**

Therefore, although both conditions are common in women of this age group, no significant association was found between AO and depressive symptoms.

Abdominal obesity (AO) is the excess adipose tissue mostly associated with metabolic risk factors, such as insulin resistance, hypertension, and dyslipidemia, which are associated with high health costs worldwide^(1)^. AO is significantly prevalent among females of all age groups developing as a growing global public health concern^(2)^. The condition notably heightens the risk of chronic non-communicable diseases (NCD), such as type 2 diabetes mellitus (T2DM) and arterial hypertension (HTA), which are particularly common in females^(3)^. The National Health and Nutrition Examination and Surveys (NHANES) reported a 20 % increase in the incidence of AO, especially in females, in the USA from 1999 to 2010^(4)^. Likewise, the reported prevalence of AO was 52·9 % in regions of Argentina, Chile and Uruguay in 2010–2011, of which a significant proportion was observed in females and was associated with a corresponding increase in the prevalence of T2DM, HTA and dyslipidemias^(5)^. In Peru, according to the Demographic and Family Health Survey (DHS), the reported prevalence of AO among men and women aged 15 years and older was 73·8 % in 2018 and 2019, while the prevalence of AO among women only was 85·1 %^(6)^. Evidently, AO is highly prevalent in the Peruvian population, especially in the female sex, which increases the risk of multiple co-morbidities.

AO in women belonging to the reproductive age group is conditioned by multiple factors, especially those associated with reproduction^(7)^. Evidence suggests that multiparity can lead to obesity and metabolic problems at any maternal age^(8)^. Other contributing factors include age, living in urban areas, type of diet and physical inactivity^(9)^. A Peruvian study noted that wealth index, level of education and living in an urban area were most associated with AO in this geographical region^(6)^. Furthermore, AO in women is known to trigger the development of concomitant health problems, such as polycystic ovary disease, hyperandrogenism, metabolic syndrome, anxiety and depression^(7,10)^. Some studies have evidenced that obese people are at greater risk of suffering from mental illnesses. Since depression is more frequent in women, they face a double risk of suffering from this disease^(11,12)^. Nevertheless, scarce studies were found in Latin America and none in Peru whose health system is segmented and deals with serious mental health problems.

Depression is a mental illness that limits one’s personal development capacities. Currently, the global incidence of depression is significantly high^(13)^ and is associated with an annual cost of one trillion dollars. However, it is estimated that if one dollar is invested in the treatment of depression, a gain of four dollars can be obtained in terms of improvements in health and work capacity^(14)^. Worldwide, there are 300 million adults with depression; the incidence increased from 172 million in 1999 to 258 million in 2017, representing a 48·8 % increase^(15)^. In Latin America, mental disorders were reported to cause disability in 34 % of people, of which, 7·8 % were attributed to depression^(16)^. In 2017, approximately 2·34 million Latin American adults were reported to suffer from depression and about 60 % of patients with this disease did not receive adequate treatment^(17)^. Based on the 2018 DHS data, 6·4 % of the Peruvian population exhibited depressive symptoms and only 14·4 % of these people received treatment for depressive symptoms^(18)^. Furthermore, compared with males, females were 2·25 times more likely to have depressive symptoms^(19)^. A significant factor that triggers the development of depression in women is intimate partner violence (IPV), which causes serious physical and mental problems^(20)^. Female victims of partner abuse, whether physical, psychological or sexual, are 2·58 times more likely to suffer from depression^(21)^. In addition, chronic diseases, such as cancer, CVD and T2DM, are also associated with a greater probability of suffering from depressive states – people with more than one chronic disease are twice as likely to suffer from depression^(10)^.

Biochemically, depressive symptoms and obesity share common non-specific indicators, that is both are characterised by an inflammatory state, increased oxidative stress and endocrine system dysfunction^(22)^. This explains why people with a higher percentage of fat are thought to have greater difficulty in achieving stabilisation of depressive symptoms^(23)^. The relationship between AO and depressive symptoms is still under investigation; however, despite substantial evidence for the Peruvian population assessing each of these variables, there are no studies focused on Peruvian women of reproductive age, who have unique characteristics and are particularly vulnerable to suffering from both conditions. Assessing this association would have implications for policymaking through the implementation of preventive measures and targeted interventions. This study aims to assess the association between AO and depressive symptoms in Peruvian women of reproductive age (18–49 years). Given the high prevalence of these clinical morbidities, it is important to gain an in-depth understanding of this potential association to develop preventive protocols, especially in the current scenario of rising prevalence. There is evidence to support the potential relationship between AO and depressive symptoms, but there have been no national analyses in the Peruvian context to examine this association. Therefore, the development of a cross-sectional study to determine the association between AO and depressive symptoms is an excellent starting point for national cohort studies focused on this association.

## Materials and methods

### Study design

In this cross-sectional observational analytical study, we examined the DHS data published in the years 2018 and 2019, a health survey conducted annually by the National Institute of Statistics and Informatics of Peru (INEI). The DHS encompasses a balanced, two-stage and probabilistic sampling with a random selection of participants from both urban and rural study areas^(24)^. This type of sampling allows for the inclusion of appropriate representative estimates of the population and replicates the population structure regarding key demographic variables, such as age and sex, among others^(24)^. Analysis of the DHS data shows representative estimates at the national level or for the total Peruvian population, as well as for the urban/rural areas, natural regions (the coast, the Andes and the Amazon) and the 25 administrative regions. The DHS data and results are publicly available and freely accessible at https://bit.ly/3OZFW0G ^(24)^.

Ethical approval for this study was obtained from the Research Ethics Committee of the Universidad Peruana de Ciencias Aplicadas (approval number: PI 060-22).

### Population

The DHS survey collected information from 6,508 clusters comprising 73 520 households, of which 29 540 belong to departmental capitals, 18 660 to urban areas and 25 320 to rural areas^(24)^. A total sample of 149 951 people was surveyed which comprised 68 259 women aged 15–49 years.

We used a pooled sample of 2 years of DHS data (2018–2019) to achieve sufficient power (>95 %). A sample size of at least 10 328 participants was estimated assuming a prevalence ratio (PR) of 1·1, an α probability of error of 0·01 and a mean exposure of 0·3 (i.e. a prevalence of 30 %) using a two-sided model and a normal distribution. The sample size was calculated using G*power 3.1.9.7.

### Data collection

The DHS participants were usual residents in the selected households or had stayed overnight the night before the interview, in case they were not residents. The survey was divided into different questionnaires – each questionnaire was directed to a specific type of resident with the understanding that not all people filled out the same questionnaire. The ‘Household Questionnaire’ collected information provided by the head of the family/spouse/a person aged >18 years who could describe the characteristics of the household members and the dwelling; their Hb sample was also taken. Next, the ‘Individual Questionnaire’ was aimed at women aged 12–49 years and collected information on their demographic and social characteristics, reproductive history and domestic violence. Finally, the ‘Health Questionnaire’ was used for all people aged ≥15 years to collect information on HTA, T2DM, mental health and anthropometric measurements^(24)^.

### Instruments and variables

#### Depressive symptoms (outcome)

Depressive symptoms (dependent variable) were defined as a set of signs and symptoms, characterised by a state of sadness, fatigue, difficulty concentrating, sleep disturbances, changes in appetite or body weight, and loss of interest or pleasure, of sufficient intensity and duration to interfere with the individual’s quality of life^(25)^. This variable was analysed as a dichotomous categorical variable and measured using the Patient Health Questionnaire (PHQ-9). The PHQ-9 is a validated tool for the early detection of depression through depressive symptoms with a reported sensitivity of 85 % and a specificity of 89 % when the cut-off point is 10^(26)^. It consists of nine questions focusing on the past 2 weeks which are based on the criteria for diagnosing clinical depression as recommended by the Diagnostic and Statistical Manual of Mental Disorders, Fifth Edition (DSM-5). Each of the nine questions is scored from 0 to 3, with a maximum score of 27^(27)^. For this study, ‘depressive symptoms’ was examined as a dichotomous variable with a cut-off value of 10 (<10: no; ≥10: yes). The cut-off value of a score of 10 on the PHQ-9 is reported to be consistent with the severity of depressive symptoms^(28)^. Additionally, the PHQ-9 has been validated in the Peruvian population with reliable sociodemographic comparisons in this population^(29)^.

#### Abdominal obesity (exposure)

The independent variable of AO was defined as abdominal circumference (in centimetres) that reflects an excess of adipose tissue associated with a high risk of contracting non-communicable diseases^(30)^. Trained anthropometrists performed measurements using a 2-m metal tape measure, after a period of exhalation and with the individual in a 2-h fasting period after eating^(31)^. This procedure was standardised according to WHO guidelines^(32)^. AO was dichotomised based on reference values (≥88 cm) established by WHO^(30)^.

#### Covariates

The following variables were analysed for the study sample: age group, educational attainment, marital status (single, married, cohabitant and separated (composed of the grouping of separated and divorced women)), natural region, area of residence, level of wealth (rich wealth level: very rich and rich groups; poor wealth level: poor and very poor groups (based on DHS categorisation)), health insurance (availing at least one of the existing health insurances at the national level (integral health insurance (SIS), social health insurance (EsSalud), police or military, and private)), smoking habit (daily cigarette smoking in the last 30 d), alcohol consumption (intake of alcoholic beverages for ≥12 d in the last year), diabetes mellitus (diagnosed by a doctor and purchases medication to control the condition), HTA (diagnosed by a physician and based on average of two blood pressure readings with systolic blood pressure of ≥140 mmHg and diastolic blood pressure or ≥90 mmHg) and IPV (defined as acts of physical, sexual, or emotional abuse by a current or former intimate male partner, and measured through the violence questionnaire aimed at women who have or have ever had a partner)^(33,34)^.

### Statistical analysis

We used the Stata® se software (version 17.0) for all analyses. Measures of central tendency (mean or median) and measures of dispersion (standard deviation or interquartile range) were used to describe the numerical variables based on their distribution. Categorical variables were described using relative and absolute frequencies. Bivariate analysis for the categorical variables was conducted using Pearson’s Chi-square test. To evaluate the association between AO and depressive symptoms, a Poisson regression analysis was employed to calculate both crude PR and adjusting ratios, taking confounding variables into account. For the adjusted model, the variables were selected using the stepwise command, and their potential collinearity was also evaluated; 95 % CI were used for all calculations. The adjusted model included variables selected using two criteria: statistical significance (AO, age, education level, marital status, natural region, alcohol, diabetes mellitus, HTA and IPV) and theoretical relevance based on a literature review (AO, age, marital status, wealth index, health insurance, alcohol, diabetes mellitus, HTA and IPV) of variables associated with the outcome (depressive symptoms).

Given the complex nature of the survey design, the ‘svy’ command in Stata® was used to weigh and reconstruct complex DHS samples. Furthermore, subpopulation analysis was included to account for the subsample obtained after applying the study selection criteria. In sensitivity analysis, we evaluated whether the characteristics of the final population were similar to those of the initial population using Pearson’s Chi-square test. The results of the sensitivity analyses are presented in see online supplementary material, Supplementary Table S1.

## Results

### Selection of study data

**Fig. 1:**
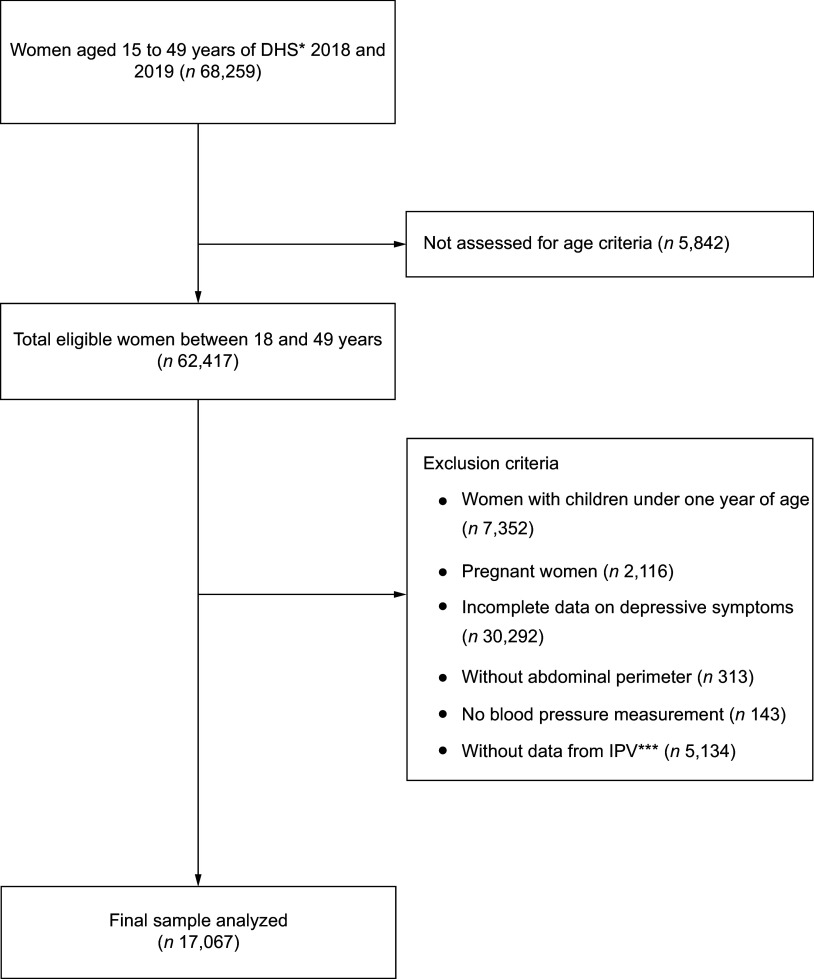
Flow chart of the study sample inclusion procedure

The initial sample included 34 971 and 33 288 records from the 2018 and 2019 DHS data, making up a total of 68 259 women aged 15–49 years who met the DHS selection criteria. Next, 5,842 women aged <18 years were excluded, resulting in a sample of 62 417 women. After applying the exclusion criteria to this sample, a total of 7,352 women were excluded because they had children under 1 year of age, 2,116 for being pregnant at the time of being surveyed and 30 748 women due to incomplete data on depressive symptoms, abdominal perimeter, or blood pressure measurements. Additionally, 5,134 women were excluded because they had no records in the IPV questionnaire^(35)^, resulting in a final sample of 17 067 women aged 18–49 years (Fig. 1). The sensitivity analysis showed that there were no significant differences between the initial and final populations for all variables except for marital status, which demonstrates that the results found uphold the representativeness of the study (see online supplementary material, Supplementary Table S1).

### Population characteristics

The most prevalent characteristics of the included women were high school as the education level (42·06 %; 95 % CI: 40·73, 43·41), cohabiting (50·86 %; 95 % CI: 49·44, 52·27) and belonging to poor socio-economic background (46·86 %; 95 % CI: 45·37, 48·35). Regarding the natural region, 30·28 % of women (95 % CI: 28·64, 31·96) lived in the Metropolitan Lima region, 28·94 % (95 % CI: 27·51, 30·40) in the Andean region and 77·02 % (95 % CI: 76·09, 77·92) in urban areas. Overall, more than half of the women suffered some type of IPV (95 % CI: 53·64 %, 56·51 %), 64·55 % (95 % CI: 63·19, 65·90) had AO and 7·61 % (95 % CI: 6·83, 8·47) were diagnosed with depressive symptoms (Table 1).

**Table 1:** General characteristics of the women (aged 18–49 years) included in the study from the DHS* 2018 and 2019 data (*n* 17 067)

Variables	Total (*n*)	%	CI 95 %
**Age (years)**			
18–29	5,874	25·10	24·01, 26·21
30–39	7,266	42·66	41·19, 44·15
40–49	3,927	32·24	30·85, 33·66
**Educational attainment**			
No education	385	2·34	2·00, 2·75
Primary	3,883	21·55	20·46, 22·68
Secondary	7,570	42·06	40·73, 43·41
Higher	5,229	34·04	32·64, 35·47
**Marital status**			
Single	188	2·25	1·66, 3·06
Married	4,333	27·51	26·19, 28·87
Cohabitant	9,778	50·86	49·44, 52·27
Widow	185	1·25	1·00, 1·56
Separate†	2,583	18·13	17·01, 19·30
**Natural region**			
Metropolitan Lima	1,807	30·28	28·64, 31·96
Rest of coast	4,864	24·93	23·74, 26·16
Andean	6,126	28·94	27·51, 30·40
Amazon	4,270	15·86	14·80, 16·97
**Area of residence**			
Urban	11 523	77·02	76·09, 77·92
Rural	5,544	22·98	22·08, 23·91
**Level of wealth** ‡			
Poor	9,940	46·86	45·37, 48·35
Middle	3,309	21·54	20·34, 22·78
Richer	3,818	31·61	30·08, 33·17
**Health insurance** §	13 886	76·92	75·63, 78·16
**Smoke** ||	75	0·56	0·35, 0·88
**Alcohol** ¶	835	7·29	6·45, 8·23
**Diabetes mellitus** **	157	1·23	0·96, 1·58
Arterial hypertension*†	1,341	9·88	8·96, 10·88
**Violence**			
Physical violence	1,931	10·17	9·37, 11·02
Sexual violence	492	2·94	2·52, 3·43
Psychological violence	8,956	53·99	52·56, 55·42
IPV*‡	9,175	55·08	53·64, 56·51
Abdominal obesity*§	10 829	64·55	63·19, 65·90
Depressive symptoms*||	1,105	7·61	6·83, 8·47

DHS, Demographic Health Survey; IPV, intimate partner violence; PHQ, Patient Health Questionnaire.

* The results were weighted considering the characteristics of the probability and two-stage sampling defined by the Peruvian DHS.

† Comprising separated and divorced women.

‡ The rich wealth level is made up of women belonging to the very rich and rich groups, and the poor wealth level is made up of the poor and very poor groups, based on the categorisation made by the DHS.

§ The woman has health insurance if she belongs to at least one of the existing health insurances at the national level (SIS, EsSalud, military and private).

|| If she smoked cigarettes daily in the last 30 d.

¶ If she consumed alcohol for ≥12 d in the last years.

** When a woman has been diagnosed by a physician and purchases medication to control the condition.

*† Whether she suffers from arterial hypertension was determined by the average of two blood pressure readings showing a systolic blood pressure reading ≥140 mmHg and a diastolic blood pressure reading ≥90 mmHg or has been diagnosed by a physician.

*‡ IPV was defined as acts of physical, sexual or emotional abuse by a current or former intimate male partner.

*§ If the abdominal circumference measurement was ≥88 cm.

*|| Assessed based on the PHQ-9 and a cut-off value of 10.

### Factors associated with depressive symptoms

In the bivariate analysis, a statistically significant association was observed between AO and depressive symptoms with 8·23 % of women with AO experiencing depressive symptoms. 9·87 % of women aged 40–49 years had depressive symptoms, while of the group of women aged 18–29 years 6·42 % had depressive symptoms. Of the cohabiting and widow women, 6·56 % and 15·29 % had depressive symptoms, respectively, and 10·62 % of the women with primary education had depressive symptoms. According to harmful behavioural habits, 11·38 % of women who consumed alcohol experienced depressive symptoms. Furthermore, 14·64 % of women with T2DM had depressive symptoms, while a 12·67 % of women with HTA had depressive symptoms. Finally, depressive symptoms were reported by 17·50 % of women who were victims of physical violence, 25·23 % of those who experienced sexual violence and 10·50 % of individuals who encountered psychological violence. All these associations were statistically significant (Table 2).

**Table 2.** Covariates and exposure variables associated with depressive symptoms in women (aged 18–49 years) included in the DHS* 2018 and 2019 data (*n* 17 067)

Variables	Depressive symptoms				*P*-value
	No depressive symptoms		With depressive symptoms		
	*n*	%	*n*	%	
Age (years)					
18–29	5,547	93·58	327	6·42	<0·001
30–39	6,827	93·40	439	6·60	
40–49	3,588	90·13	339	9·87	
**Educational attainment**					
No education	358	92·05	27	7·95	<0·001
Primary	3,571	89·38	312	10·62	
Secondary	7,069	92·01	501	7·99	
Higher	4,964	94·80	265	5·20	
**Marital status**					
Single	170	91·63	18	8·37	<0·001
Married	4,107	92·99	226	7·01	
Cohabitant	9,223	93·44	555	6·56	
Widow	155	84·71	30	15·29	
Separate†	2,307	89·17	276	10·83	
**Natural region**					
Metropolitan Lima	1,675	91·61	132	8·39	0·091
Rest of coast	4,597	93·28	267	6·72	
Andean	5,657	91·55	469	8·45	
Amazon	4,033	94·04	237	5·96	
**Area of residence**					
Urban	10 767	92·21	756	7·79	0·254
Rural	5,195	93·01	349	6·99	
**Level of wealth** ‡					
Poor	9,246	92·01	694	7·99	0·613
Middle	3,098	92·39	211	7·61	
Richer	3,618	92·96	200	7·04	
**Health insurance** §					
Yes	13 006	92·45	880	7·55	0·794
No	2,956	92·19	225	7·81	
**Smoke** ||					
Yes	64	90·17	11	9·83	0·539
No	15 898	92·40	1,094	7·60	
**Alcohol** ¶					
Yes	745	88·62	90	11·38	0·029
No	15 217	92·69	1,015	7·31	
**Diabetes mellitus** **					
Yes	130	85·36	27	14·64	0·016
No	15 832	92·48	1,078	7·52	
Arterial hypertension*†					
Yes	1,199	87·33	142	12·67	<0·001
No	14 763	92·95	963	7·05	
Violence*‡					
Physical					
Yes	1,612	82·50	319	17·50	<0·001
No	14 350	93·51	786	6·49	
Sexual					
Yes	364	74·77	128	25·23	<0·001
No	15 598	92·93	977	7·07	
Psychological					
Yes	8,120	89·50	836	10·50	<0·001
No	7,842	95·78	269	4·22	
Abdominal obesity*§					
Yes	10 109	91·77	720	8·23	0·027
No	5,863	93·52	385	6·48	

DHS, Demographic Health Survey; IPV, intimate partner violence.

* The results were weighted considering the characteristics of the probability and two-stage sampling defined by the Peruvian DHS.

† Composed of separated and divorced women.

‡ The rich wealth level is made up of women belonging to the very rich and rich groups and the poor wealth level is made up of the poor and very poor groups, based on the categorisation made by the DHS.

§ The woman has health insurance if she belongs to at least one of the existing health insurances at the national level (SIS, EsSalud, military and private).

|| If she smoked cigarettes daily in the last 30 d.

¶ If she consumed alcohol ≥12 d in the last year.

** Occurs when a woman has been diagnosed by a physician and purchases medication to control the condition.

*† Whether she suffers from arterial hypertension was determined by the average of two blood pressure readings showing a systolic blood pressure reading ≥140 mmHg and a diastolic blood pressure reading ≥90 mmHg or has been diagnosed by a physician.

*‡ IPV was defined as acts of physical, sexual or emotional abuse by a current or former intimate male partner.

*§ Abdominal circumference measurement ≥88 cm.

### Multivariable analysis between abdominal obesity and depressive symptoms

Table 3 presents the results of the crude and adjusted regression analysis examining the association between AO and depressive symptoms. In the adjusted statistical model, the prevalence of depressive symptoms was 12 % higher in women with AO compared to those without AO; however, this result was not statistically significant (PR = 1·12, 95 % CI: 0·91, 1·38, *P* = 0·282). Similarly, the adjusted theoretical model showed that women with AO had a 15 % higher prevalence of depressive symptoms compared to those without AO (PR = 1·15, 95 % CI: 0·92, 1·42); this difference was not statistically significant (*P* = 0·213).

**Table 3.** Results of regression analysis between depressive symptoms and abdominal obesity in women aged 18–49 years in the DHS* 2018 and 2019 data (*n* 17 067)

Variable	Depressive symptoms								
	Raw PR			Adjusted statistical model†			Adjusted theoretical model‡		
	PR	95 % CI	*P*-value	PR	95 % CI	*P*-value	PR	95 % CI	*P*-value
**Abdominal obesity**									
No abdominal obesity	Ref			Ref			Ref		
With abdominal obesity	1·26	1·03, 1·57	0·028	1·12	0·91, 1·38	0·282	1·15	0·92, 1·42	0·213
**Age (years)**									
30–39				0·96	0·74, 1·25	0·775	0·99	0·76, 1·31	0·975
40–49				1·22	0·92, 1·63	0·172	1·33	0·99, 1·78	0·056
**Educational attainment**									
Primary				1·48	0·87, 2·53	0·151			
Secondary				1·10	0·64, 1·89	0·726			
Higher				0·71	0·40, 1·27	0·247			
**Marital status**									
Married				0·99	0·47, 2·07	0·979	1·05	0·49, 2·23	0·896
Cohabitant				0·89	0·44, 1·81	0·762	0·95	0·46, 1·97	0·900
Widow				1·64	0·71, 3·79	0·244	1·82	0·79, 4·23	0·162
Separate				1·22	0·59, 2·52	0·581	1·23	0·58, 2·58	0·586
**Natural region**									
Rest of coast				0·74	0·53, 1·01	0·061			
Sierra				0·88	0·65, 1·19	0·409			
Forest				0·67	0·48, 0·93	0·018			
**Alcohol**									
Yes				1·55	1·04, 2·29	0·028	1·47	0·99, 2·19	0·053
**Diabetes mellitus**									
Yes				1·76	0·98, 3·16	0·060	1·60	0·89, 2·88	0·115
**Arterial hypertension**									
Yes				153	1·13, 2·08	0·07	1·54	1·12, 2·10	0·007
**IPV**									
Yes				2·21	1·73, 2·82	<0·001	2·32	1·82, 2·96	<0·001
**Level of wealth**									
Middle							0·87	0·66, 1·14	0·326
Richer							0·80	0·59, 1·08	0·150
**Area of residence**									
Rural							0·89	0·72, 1·09	0·249
**Health insurance**									
Yes							0·96	0·75, 1·23	0·760

DHS, Demographic Health Survey; PR, prevalence ratio; IPV, intimate partner violence.

* The results were weighted considering the characteristics of the probability and two-stage sampling defined by the Peruvian DHS.

† Adjusted for abdominal obesity, age, education level, marital status, natural region, alcohol, diabetes mellitus, arterial hypertension and IPV.

‡ Adjusted for abdominal obesity, age, marital status, wealth index, health insurance, alcohol, diabetes mellitus, arterial hypertension and IPV.

## Discussion

### Main findings and interpretations

We found a significant association between AO and depressive symptoms in the crude regression analysis, which indicated that women with AO had a 26 % increased risk of experiencing depressive symptoms. However, when other covariates were included in the multivariable model, the association was not statistically significant. Other studies from Mexico and the Netherlands^(36,37)^ reported higher odds of having depressive symptoms among women with elevated total body fat, abdominal adiposity, BMI and waist circumference. In this regard, a WHO report reiterated that women with AO have a very high risk of suffering from metabolic diseases when they present a BMI > 30 kg/m^2^, that is, obese women, while for overweight women (BMI = 25–29·99 kg/m^2^) this risk is lower^(30)^. It has been shown that abdominal circumference may be more variable than BMI over time because the abdominal circumference may differ for the same BMI values^(38,39)^. Thus, there might be some intricate factors inherent to the study design or to the Peruvian population that would explain our results.

### Comparison with other studies

Our results concur with those reported by Zavala *et al.* ^(36)^ who examined the Mexican population and concluded that there was no association between AO and depression in both sexes when the model was adjusted for other variables, such as age, having a partner, presence of diabetes and educational level. Despite this, they described a statistically significant association between waist circumference measurement and the depression score for women – as the waist circumference increased by one, the depressive symptom score increased by 0·05. However, a significant increase in abdominal girth measurement was required to move up one point in the depressive symptoms score, which supports the above-mentioned results. Likewise, Luo *et al.* ^(2)^ evaluated the same association in the Chinese population and found that no statistically significant association exists between AO and depression in women, even though they had a high prevalence of these two conditions. We also did not observe any significant association between AO and depression in women; however, women did tend to have a higher prevalence of depression in women compared to men.

Several studies have supported the existence of the association between AO and depression and have suggested to be explained by biological, environmental and lifestyle factors^(40)^. Solomon *et al.* ^(41)^ described that women are more prone to suffer depression during periods of greater hormonal fluctuations in their life, such as the premenstrual and perimenopausal periods, mainly because hormones such as estradiol and progesterone, that can regulate the mood and the hypothalamic–pituitary–adrenocortical axis. On the other hand, research targeting obese individuals has shown that there is an elevated production of pro-inflammatory cytokines in obesity which is capable of producing mood disorders through the regulation of tryptophan, an amino acid precursor in the production of serotonin^(42)^. Obesity and depression reportedly have a bidirectional relationship, wherein depressive symptoms promote the development of metabolic syndrome, which as a pathology is closely linked to AO^(43)^. Although these are all plausible causal explanations, our study did not find a significant relationship after adjusting for multiple confounding variables, which did not include the factors mentioned in this paragraph.

### Public health implications

Although the association between AO and depressive symptoms was not significant, it is noteworthy that both conditions harm women’s health and quality of life; therefore, appropriate strategies must be formulated to reduce the disease burden of these conditions which can be achieved through health education, early detection and timely intervention^(38,44)^. Regarding obesity, it is believed that 21 % of women will be obese worldwide; however, AO is often not considered in this context despite waist circumference being a better predictor of cardiometabolic diseases and can help identify people with metabolic problems at an earlier stage^(44)^. There is strong evidence suggesting that obesity and depression coexist, and adequate treatment of either one can bring about significant improvement in the other condition^(45)^. Therefore, comprehensive patient care should consider the detection and treatment of both diseases should the patient present with either condition.

### Limitations and strengths

Our study has methodological limitations that should be taken into account. First, due to the cross-sectional design, it was not possible to establish causal relationship between AO and depressive symptoms. Also, there could be retro causality as the depressive disorder is associated with eating disorders. Second, this study was based on secondary data obtained from a national-level survey. Therefore, there is a possibility of inaccuracy in the records due to memory bias on the part of the respondents. Nevertheless, the data present in the survey are the closest approximation to the reality of the participants, and it was collected by trained pollsters. Third, it was not possible to clinically diagnose depression in the participants since the instrument used in the DHS only measured depressive symptoms in the last 2 weeks; therefore, we used the validated PHQ-9 questionnaire that helped to identify depressive symptoms that are comparable to an early diagnosis of depression^(29)^. Likewise, the measurement of AO was done by a trained evaluator which reduced measurement biases, in addition to validating its use as an indirect indicator of AO^(46)^. Fourth, it is possible that there is a selection bias, since a significant number of women who did not have complete data on the variables of interest were eliminated; however, the sensitivity analysis shows that the included population maintains most of the characteristics of the initial population. Therefore, we believe that the results should not change significantly after applying our inclusion criteria. Despite the above limitations, this study included significant data from a large representative national-level survey (DHS) of the population. Validated and standardised instruments were used for each variable, as well as the standard DHS model^(47)^, which allowed us to better understand the health status of Peruvian women. Moreover, to the best of our knowledge, this is the first study analysing this variable in the Peruvian setting.

## Conclusions

Both AO and depressive symptoms are very common in Peruvian women of reproductive age. According to the data analysed, no association was found between AO and depressive symptoms. Future studies should evaluate these variables prospectively, taking into account the factors associated with plausible explanations.

## Supporting information

Leon-Zamora et al. supplementary material Leon-Zamora et al. supplementary material

## Supplementary material

For supplementary material accompanying this paper visit https://doi.org/10.1017/S1368980024000867
