# Supplementary material for: Association between abdominal obesity and depressive symptoms in Peruvian women aged 18–49 years: a sub-analysis of the Demographic and Family Health Survey 2018–2019
Source: Public Health Nutr. 2024 Apr 12;27(1):e114. doi: 10.1017/S1368980024000867 (PMC11036433; doi:10.1017/S1368980024000867)
Supplement: Leon-Zamora et al. supplementary material [file S1368980024000867sup001.docx]

**7. Supplementary Materials:**

**Table S1.** Sensitivity analysis of the DHS^1^ 2018 and 2019 data

| **Variables** | **PI^2^ (62,417)** | **PF^3^ (17,067)** | ***p-*value** |
| --- | --- | --- | --- |
|  | ***n* (%)** | ***n* (%)** |  |
| **Age (years)** |  |  |  |
| 18–29 | 29,695 (39.04) | 5,874 (25.10) | 0.099 |
| 30–39 | 22,102 (32.58) | 7,266 (42.66) |  |
| 40–49 | 13,620 (28.39) | 3,927 (32.24) |  |
| **Educational attainment** |  |  |  |
| No education | 1,285 (1.74) | 27 (2.34) | 0.638 |
| Primary | 12,441 (16.81) | 3,883 (21.55) |  |
| Secondary | 26,305 (39.07) | 7,570 (42.06) |  |
| Higher | 22,386 (42.39) | 5,229 (34.04) |  |
| **Marital status** |  |  |  |
| Single | 10,537 (26.14) | 188 (2.25) | <0.001 |
| Married | 13,958 (22.00) | 4,333 (27.51) |  |
| Cohabitant | 30,550 (38.92) | 9,778 (50.86) |  |
| Widow | 474 (0.84) | 185 (1.25) |  |
| Separate^4^ | 6,898 (12.11) | 2,583 (18.13) |  |
| **Natural region** |  |  |  |
| Metropolitan Lima | 8,536 (37.36) | 1,807 (30.28) | 0.689 |
| Rest of coast | 19,179 (25.48) | 4,864 (24.93) |  |
| Andean | 20,344 (24.35) | 6,126 (28.94) |  |
| Amazon | 14, 358 (12.80) | 4,270 (15.86) |  |
| **Area of residence** |  |  |  |
| Urban | 44,838 (81.91) | 11,197 (77.02) | 0.394 |
| Rural | 17,579 (18.09) | 5,544 (22.98) |  |
| **Level of wealth^5^** |  |  |  |
| Poor | 31,869 (37.63) | 9,940 (46.86) | 0.338 |
| Middle | 12,935 (21.60) | 3,309 (21.54) |  |
| Richer | 17,613 (40.77) | 3,818 (31.61) |  |
| **Health insurance^6^** |  |  |  |
| Yes | 49,083 (72.56) | 10,366 (76.92) | 0.503 |
| **No** | 13,334 (27.44) | 3,181 (23.08) |  |
| **Smoke^7^** |  |  |  |
| Yes | 120 (0.23) | 75 (0.56) | 0.138 |
| **No** | 62,297 (99.77) | 16,992 (99.44) |  |
| **Alcohol^8^** |  |  |  |
| Yes | 1,432 (3.80) | 835 (7.29) | 0.281 |
| **No** | 60,985 (96.20) | 16,232 (92.71) |  |
| **Diabetes mellitus^9^** |  |  |  |
| Yes | 232 (1.13) | 157 (1.23) | 0.948 |
| **No** | 26,557 (98.87) | 16,910 (98.77) |  |
| **Arterial hypertension^10^** |  |  |  |
| Yes | 1,863 (8.44) | 1,341 (9,88) | 0.724 |
| **No** | 24,582 (91.56) | 15,726 (90.12) |  |
| **Violence^11^** |  |  |  |
| Physical |  |  |  |
| Yes | 4,575 (9.81) | 1,931 (10.17) | 0.932 |
| No | 30,170 (90.19) | 15,136 (89.83) |  |
| Sexual |  |  |  |
| Yes | 1,123 (2.57) | 492 (2.94) | 0.873 |
| No | 42,622 (97.43) | 16,575 (97.06) |  |
| Psychological |  |  |  |
| Yes | 22,179 (52.34) | 8,959 (53.99) | 0.815 |
| No | 21,566 (47.66) | 8,111 (46.01) |  |

^1^The results were weighted considering the characteristics of the probability and two-stage sampling defined by the Peruvian Demographic Health Survey (DHS); ^2^Initial population of women aged 15 to 49 years; ^3^Final population of women aged 18 to 49 years to whom the exclusion criteria were applied; ^4^Composed of the grouping of separated and divorced women; ^5^The rich wealth level is made up of women belonging to the very rich and rich groups and the poor wealth level is made up of the poor and very poor groups, based on the categorization made by the DHS; ^6^If she belongs to at least one of the existing health insurances at the national level (SIS, EsSalud, military, private); ^7^A woman is considered to be a smoker if she smoked cigarettes daily in the last 30 days; ^8^A woman is considered to be consuming alcoholic beverages if she consumed alcohol for ≥12 days in the last year; ^9^Occurs when a woman has been diagnosed by a physician and purchases medication to control the condition; ^10^Suffer from arterial hypertension if the average of two blood pressure readings shows a systolic blood pressure ≥140 mmHg and a diastolic blood pressure ≥90 mmHg, or has been diagnosed by a physician; ^11^A woman is considered to be a sufferer of violence if she is the victim of acts of physical, sexual or emotional abuse by her current or former partner.

**Table S2.** Variables associated with abdominal obesity in women aged 18–49 years participating in the DHS^1^ 2018 and 2019 (n = 17 067).

| **Variables** | **Abdominal obesity** | | ***p-*value** |
| --- | --- | --- | --- |
|  | **No abdominal obesity** | **Abdominal obesity** |  |
|  | ***n* (%)** | ***n* (%)** |  |
| **Age (years)**  18–29  30–39  40–49 |  |  |  |
|  | 2,892 (51.06) | 2,982 (48,94) | <0.001 |
|  | 2,336 (35.10) | 4,930 (64.90) |  |
|  | 1,010 (23.74) | 2,917 (76.26) |  |
| **Educational attainment** |  |  |  |
| No education | 153 (34,89) | 232 (65.11) | < 0.001 |
| Primary | 1,377 (31,81) | 2,506 (68.19) |  |
| Secondary | 2,737 (33.35) | 4,833 (66.65) |  |
| Higher | 1,971 (40.37) | 3,258 (59.63) |  |
| **Marital status** |  |  |  |
| Single | 87 (44.37) | 101 (55.63) | 0.013 |
| Married | 1,436 (32.36) | 2,897 (67.64) |  |
| Cohabitant | 3,619 (35.47) | 6,159 (64,53) |  |
| Widow | 67 (35.17) | 118 (64.83) |  |
| Separate^2^ | 1,029 (38.96) | 1,554 (61.04) |  |
| **Natural region** |  |  |  |
| Metropolitan Lima | 615 (33.91) | 1,192 (66.09) |  |
| Rest of coast | 1,419 (29.38) | 3,445 (70.62) | <0.001 |
| Andean | 2,511 (39.80) | 3,615 (69.20) |  |
| Amazon | 1,693 (39.97) | 2,577 (60.03) |  |
| **Area of residence** |  |  |  |
| Urban | 3,872 (33.52) | 7,651 (66.48) | <0.001 |
| Rural | 2,366 (41.91) | 3,178 (58.09) |  |
| **Level of wealth^3^** |  |  |  |
| Poor | 3,884 (37.13 | 6,056 (62.87) | 0.014 |
| Middle | 1,029 (31.48) | 2,280 (68.52) |  |
| Richer | 1,325 (35.66) | 2,493 (64.34) |  |
| **Health insurance^4^** |  |  |  |
| Yes | 5,100 (35.46) | 8,786 (64.54) | 0.975 |
| No | 1,138 (35.40) | 2,043 (64.60) |  |
| **Smoke^5^** |  |  |  |
| Yes | 22 (33.41) | 53 (66.59) | 0.848 |
| No | 6,216 (35.46) | 10,776 (64.54) |  |
| **Alcohol^6^** |  |  |  |
| Yes | 292 (38.06) | 543 (61.94) | 0.392 |
| No | 5,946 (35.24) | 10,286 (64.76) |  |
| **Diabetes mellitus^7^** |  |  |  |
| Yes | 28 (12.94) | 129 (87.06) | <0.001 |
| No | 6,210 (35.73) | 10,700 (64.27) |  |
| **Arterial hypertension^8^** |  |  |  |
| Yes | 282 (18.95) | 1,059 (81.05) | <0.001 |
| No | 5,956 (37.25) | 9,770 (62.75) |  |
| **Violence^9^** |  |  |  |
| Physical |  |  |  |
| Yes | 767 (38.07) | 1,164 (61.93) | 0.150 |
| No | 5,471 (35.15) | 9,665 (64.85) |  |
| Sexual |  |  |  |
| Yes | 197 (43.28) | 295 (56.72) | 0.042 |
| No | 6,041 (35.21) | 10,534 (64.79) |  |
| Psychological |  |  |  |
| Yes | 3,343 (34.86) | 5,613 (65.14) | 0.314 |
| No | 2,895 (36.13) | 5,216 (63.87) |  |
| **Depressive Symptoms^10^** |  |  |  |
| Yes | 385 (30.21) | 720 (69.79) | 0.027 |
| No | 5,853 (35.88) | 10,109 (64.12) |  |

^1^The results were weighted considering the characteristics of the probability and two-stage sampling defined by the Peruvian Demographic Health Survey (DHS); ^2^ Composed of separated and divorced women; ^3^The rich wealth level is made up of women belonging to the very rich and rich groups and the poor wealth level is made up of the poor and very poor groups, based on the categorization made by the DHS; ^4^ The woman has health insurance if she belongs to at least one of the existing health insurances at the national level (SIS, EsSalud, military, private); ^5^If she smoked cigarettes daily in the last 30 days; ^6^If she consumed alcohol for ≥12 days in the last year; ^7^Occurs when a woman has been diagnosed by a physician and purchases medication to control the condition; ^8^If suffer from arterial hypertension if the average of two blood pressure readings shows a systolic blood pressure ≥140 mmHg and a diastolic blood pressure ≥90 mmHg, or has been diagnosed by a physician; ^9^ Intimate Partner Violence (IPV) was defined as acts of physical, sexual, or emotional abuse by a current or former intimate male partner; ^10^Assessed based on the PHQ-9 and a cut-off point of 10.
